# In Vitro Cell Sensitivity to Palytoxin Correlates with High Gene Expression of the Na^+^/K^+^-ATPase β2 Subunit Isoform

**DOI:** 10.3390/ijms21165833

**Published:** 2020-08-14

**Authors:** Marco Pelin, Gabriele Stocco, Chiara Florio, Silvio Sosa, Aurelia Tubaro

**Affiliations:** Department of Life Sciences, University of Trieste, 34127 Trieste, Italy; mpelin@units.it (M.P.); stoccog@units.it (G.S.); florioc@units.it (C.F.); ssosa@units.it (S.S.)

**Keywords:** palytoxin, Na^+^/K^+^-ATPase, toxicogenetic, cytotoxicity, genetic variants

## Abstract

The marine polyether palytoxin (PLTX) is one of the most toxic natural compounds, and is involved in human poisonings after oral, inhalation, skin and/or ocular exposure. Epidemiological and molecular evidence suggest different inter-individual sensitivities to its toxic effects, possibly related to genetic-dependent differences in the expression of Na^+^/K^+^-ATPase, its molecular target. To identify Na^+^/K^+^-ATPase subunits, isoforms correlated with *in vitro* PLTX cytotoxic potency, sensitivity parameters (EC_50_: PLTX concentration reducing cell viability by 50%; Emax: maximum effect induced by the highest toxin concentration; 10^−7^ M) were assessed in 60 healthy donors’ monocytes by the MTT (methylthiazolyl tetrazolium) assay. Sensitivity parameters, not correlated with donors’ demographic variables (gender, age and blood group), demonstrated a high inter-individual variability (median EC_50_ = 2.7 × 10^−10^ M, interquartile range: 0.4–13.2 × 10^−10^ M; median Emax = 92.0%, interquartile range: 87.5–94.4%). Spearman’s analysis showed significant positive correlations between the β2-encoding ATP1B2 gene expression and Emax values (rho = 0.30; *p* = 0.025) and between Emax and the ATP1B2/ATP1B3 expression ratio (rho = 0.38; *p* = 0.004), as well as a significant negative correlation between Emax and the ATP1B1/ATP1B2 expression ratio (rho = −0.30; *p* = 0.026). This toxicogenetic study represents the first approach to define genetic risk factors that may influence the onset of adverse effects in human PLTX poisonings, suggesting that individuals with high gene expression pattern of the Na^+^/K^+^-ATPase β2 subunit (alone or as β2/β1 and/or β2/β3 ratio) could be highly sensitive to PLTX toxic effects.

## 1. Introduction

Palytoxin (PLTX) is one of the most potent marine toxins, originally identified in *Palythoa* zoanthids corals [1]. Subsequently, PLTX and/or its analogs, among which are ovatoxin-a (OVTX-a), have been detected in *Ostreopsis* dinoflagellates [2,3,4,5,6,7,8,9,10] and in marine cyanobacteria [11]. In the last three decades, blooms of *Ostreopsis* cf. *ovata* have been frequently reported in temperate areas, such as the Mediterranean Sea and the Atlantic coasts of Portugal, and were often associated with adverse effects in the respiratory tract, eyes, and skin [12,13,14,15,16]. In addition, increasing reports of adverse effects after inhalational and/or cutaneous exposure to water and/or vapors from aquaria containing *Palythoa* and *Zoanthus* corals—widely used as decorative elements—are documented worldwide [17,18]. On the other hand, the main problem in tropical areas is represented by PLTX accumulation in edible marine organisms, the consumption of which has been associated with a series of severe human poisonings, sometimes with fatal outcomes. The major symptoms of these foodborne poisonings are gastrointestinal distress, myalgia, and muscular cramps, impaired cardiac functions, dyspnoea and respiratory failure sometimes leading to death [14,19,20].

Some evidence in the documented cases of human poisonings ascribed to PLTXs, especially the foodborne ones, suggest a high inter-individual variability to PLTX’s toxic effects. Indeed, in 1987 a man and a woman were poisoned after consumption of the same parrotfish (*Scarus ovifrons*) contaminated by PLTX but the man recovered within one week, while the woman died after 4 days due to respiratory failure, associated with muscular damage [21]. Similarly, in 1994 a woman died the day after eating a PLTX-contaminated tropical sardine (*Herklotsichthys quadrimaculatus*), while her son did not develop any symptoms [22]. In 2002, only 11 people out of 33 that ate the same meal (PLTX-contaminated serranid fish, *Epinephelus bruneus*) developed symptoms of poisoning and 7 of them required hospitalization [23]. Differences in inter-individual sensitivities can be noticed also considering the documented cases of poisonings associated with inhalational exposure to vapors generated during the cleaning procedures of aquaria containing soft corals contaminated by PLTXs. In particular, in a poisoning episode occurred in The Netherlands, 4 patients were exposed at the same time to vapours coming from the same home aquarium containing PLTX-contaminated soft corals but only two of them developed several symptoms (i.e., dyspnoea and fatigue) persisting for few months [24].

Even though these different inter-individual toxic responses may be due to several factors, such as concomitant pathologies or different exposure levels, a genetic-based variable individual sensitivity to PLTX could be hypothesized. This hypothesis is supported by the existence of genetic variants of the molecular target of PLTX, the Na^+^/K^+^-ATPase, converted by the toxin in an unspecific cationic channel [25,26]. The Na^+^/K^+^-ATPase is a proteic heterocomplex composed by one α and one β subunit together with an additional γ regulatory protein. Each of the α and the β subunits present more than one isoform, encoded by different genes, with different tissues expression pattern, as reported by the Model Organism Protein Expression Database (MOPED) [27]. Moreover, evidence demonstrates different expression patterns of Na^+^/K^+^-ATPase subunit isoforms between males and females [28,29,30] and it is well known that some isoforms of the α and β subunits own different affinities and sensitivities towards cardioactive glycosides [31,32,33,34,35]. Intriguingly, cardioactive glycosides, such as ouabain, are reported to inhibit PLTX *in vitro* effects [36,37,38,39,40], being able to modulate PLTX action on the Na^+^/K^+^-ATPase in a complex manner: Ouabain acts as a negative allosteric modulator against high PLTX concentrations and as a non-competitive antagonist against low PLTX concentrations [41].

On the basis of these observations, this study was carried out following a toxicogenetic approach to identify the α and β Na^+^/K^+^-ATPase subunits isoforms correlated with the *in vitro* cell sensitivity to PLTX. The results would provide a contribution to identify genetic risk factors for the toxic outcomes.

## 2. Results

### 2.1. Healthy Volunteers

Sixty healthy adult volunteers, with no ongoing drug treatments or pathologies, were enrolled. For each volunteer, data related to gender, age and blood group were recorded. Of the 60 volunteers, 31.7% were female and 68.3% male (19 and 41, respectively), the median age was 42.0 years (interquartile range: 30.5–51.8) and the major blood groups were 5% 0^−^, 36% 0^+^, 8% A^−^, 20% A^+^, 7% B^−^, 5% B^+^, 2% AB^−^, and 17% AB.

### 2.2. PLTX In Vitro Sensitivity

**Figure 1:**
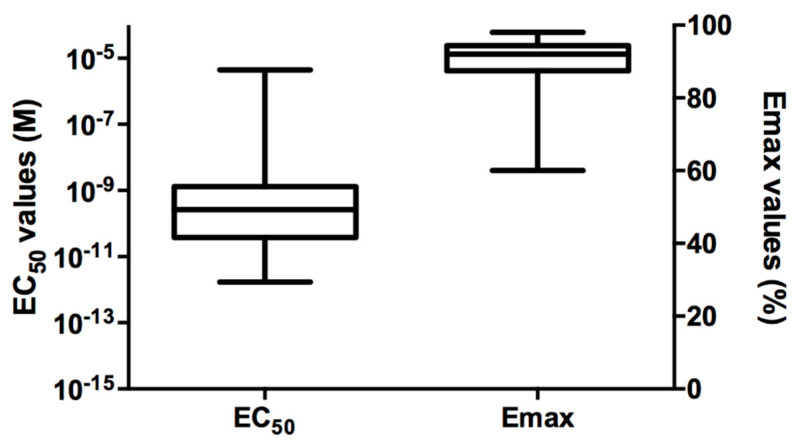
Distribution of EC_50_ and Emax values measured by the MTT assay in monocytes from the 60 healthy volunteers exposed to PLTX for 4 h. For the monocytes of each volunteer, EC_50_ and Emax values were the means of three experiments performed in triplicate.

For each volunteer, monocyte sensitivity to PLTX was assessed by cytotoxicity (methylthiazolyl tetrazolium, MTT, assay) and expressed as EC_50_ and Emax values. Figure 1 shows the distribution of these sensitivity parameters within the 60 healthy volunteers enrolled in the study. The median EC_50_ value was equal to 2.7 × 10^−10^ M (interquartile range: 0.4–13.2 × 10^−10^ M), while the median Emax value was 92.0% (interquartile range 87.5–94.4%). In general, the distribution analysis of the sensitivity parameters (EC_50_ and Emax) highlighted a wide variability of the individual in vitro monocyte sensitivity to PLTX within the 60 healthy volunteers.

### 2.3. Genetic Analysis: Expression of the Na^+^/K^+^-ATPaseαand β Subunits Isoforms

**Figure 2:**
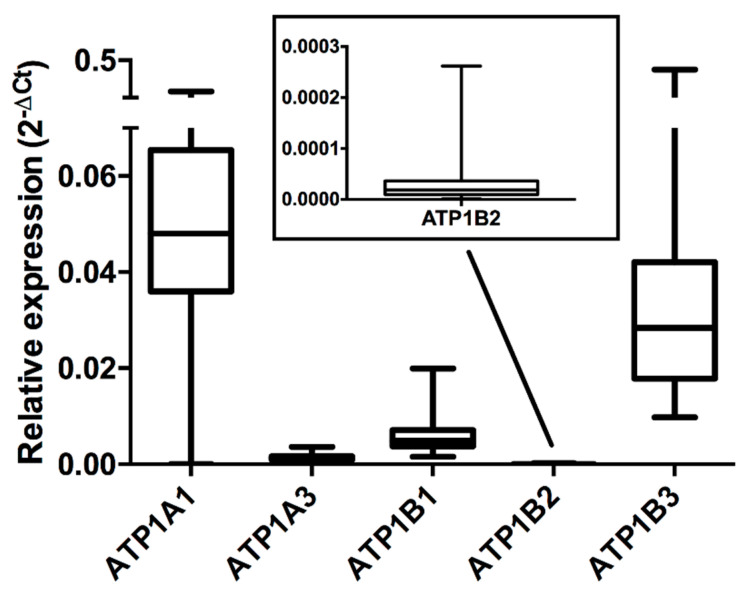
Distribution of relative gene expressions for the Na^+^/K^+^-ATPase α subunit isoforms (ATP1A1, ATP1A3) and β subunit isoforms (ATP1B1, ATP1B2 and ATP1B3) calculated as 2^−ΔCt^ with respect to the housekeeping ACTB gene within monocytes from the 60 healthy volunteers enrolled in the study. For each volunteer, relative expression data were the means of three experiments performed in duplicate.

The distribution of the relative expressions of the genes encoding for the α (ATP1A1 and ATP1A3) and β (ATP1B1, ATP1B2 and ATP1B3) subunits isoforms of the Na^+^/K^+^-ATPase in monocytes from the 60 healthy volunteers is shown in Figure 2. In particular, for the α subunit, the median gene expressions of the α1 isoform (ATP1A1) and the α3 isoform (ATP1A3) were equal to 0.0480 (interquartile range: 0.0360–0.0654) and 0.0013 (interquartile range: 0.0009–0.0016), respectively. For the β isoforms, the median gene expressions of β1 (ATP1B1), β2 (ATP1B2), and β3 (ATP1B3) were equal to 0.0048 (interquartile range: 0.0038–0.0071), 1.87 × 10^−5^ (interquartile range: 0.98−3.67 × 10^−5^) and 0.0284 (interquartile range 0.0178–0.0421), respectively. Additionally, in this case, results show a large inter-individual variability in the gene expression for these isoforms among the monocytes from the 60 healthy volunteers.

Relative expression data of the Na^+^/K^+^-ATPase isoforms of male volunteers (68.3%) were compared to those of female volunteers (31.7%; Supplementary Figure S1). For the α isoforms, the median relative gene expressions of α1 (ATP1A1) in males and females were equal to 0.0481 (interquartile range: 0.0383–0.0649) and 0.0412 (interquartile range: 0.0336–0.0688), respectively, whereas the median relative gene expressions of α3 (ATP1A3) in males and females were equal to 0.0013 (interquartile range: 0.0009–0.0018) and 0.0013 (interquartile range: 0.0009–0.00145), respectively. Considering the β isoforms, the median relative gene expressions of β1 isoform (ATP1B1) in males and females were equal to 0.0049 (interquartile range: 0.0037–0.0072) and 0.0045 (interquartile range: 0.0040–0.0071), respectively; for the β2 isoform (ATP1B2) the median relative gene expression was 1.61 × 10^−5^ for males (interquartile range: 0.60–3.26 × 10^−5^) and 2.22 × 10^−5^ for females (interquartile range: 1.14–3.96 × 10^−5^), whereas, the median relative gene expression for the β3 isoform (ATP1B3) was 0.0299 for males (interquartile range: 0.0188–0.0437) and 0.0212 for females (interquartile range: 0.0148–0.0408). For all the isoforms considered, the non-parametric analysis for non-normalized unpaired data (Mann–Whitney test) showed no significant difference (*p* > 0.05) between males and females, suggesting the absence of differences in the gene expression of Na^+^/K^+^-ATPase isoforms between genders. In addition, the non-parametric Spearman’s correlation analysis between the relative gene expressions of the Na^+^/K^+^-ATPase isoforms and age of the enrolled healthy volunteers showed a significant negative correlation only for the relative expression of the ATP1B3 gene (rho = −0.30; *p* = 0.021), suggesting that as the age increases, ATP1B3 expression decreases. On the contrary, no significant correlations were determined by the non-parametric correlation analysis between age and the relative gene expressions of the other isoforms.

Finally, it was assessed whether the blood group could be related to a different pattern of gene expression of the Na^+^/K^+^-ATPase subunits isoforms (Supplementary Figure S2). The analysis for unpaired data (Kruskal–Wallis test) followed by the Dunn’s post-test did not demonstrate any significant difference (*p* > 0.05), suggesting no relation between blood group and the expression of the Na^+^/K^+^-ATPase subunits isoforms.

### 2.4. Correlation between In Vitro Cells Sensitivity to PLTX and Demographic Variables

**Figure 3:**
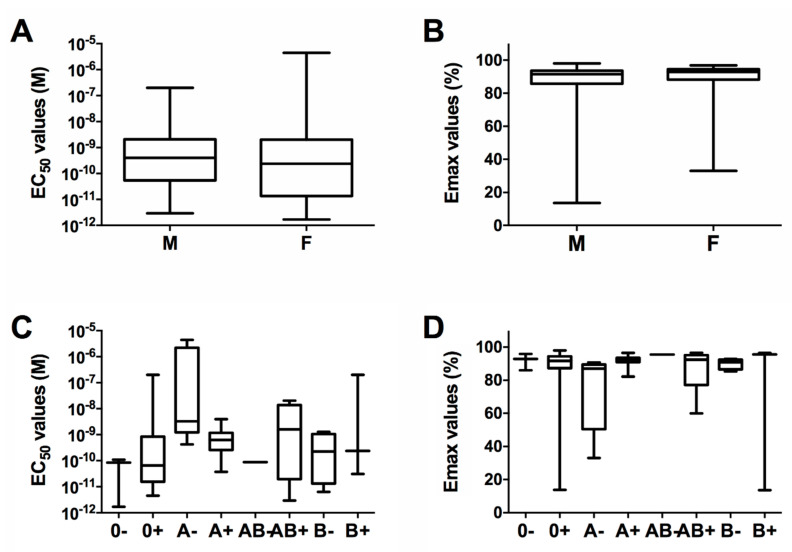
Distribution of EC_50_ (**A**,**C**) and Emax (**B**,**D**) values measured by the MTT assay in monocytes from the 60 healthy volunteers exposed to PLTX from 4 h, within genders (male = 41; female = 19) (A,B) or blood groups (0^−^ = 3; 0^+^ = 22; A^−^ = 5; A^+^ = 12; AB^−^ = 1; AB^+^ = 10; B^−^ = 4; B^+^ = 3) (panels C,D). For each volunteer, EC_50_ and Emax values were the means of three experiments performed in triplicate.

To analyze whether the demographic data of each healthy volunteer (gender, age, and blood group) are related to differences in their monocytes sensitivity to PLTX, *in vitro* cytotoxicity data (EC_50_ and Emax) were compared to the selected population parameters. Considering the gender, sensitivity values were grouped between males and females and the relevant distributions of EC_50_ and Emax values are shown in Figure 3A,B. The median EC_50_ values for males and females were equal to 4.0 × 10^−10^ M (interquartile range: 0.5–20.9 × 10^−10^ M) and 2.4 × 10^−10^ M (interquartile range: 0.1–20.3 × 10^−10^ M), respectively. The median Emax values for males and females were 91.5% (interquartile range: 87.8%–93.7%) and 92.9% (interquartile range: 88.2%–94.6%), respectively. The non-parametric analysis for unpaired data (Mann–Whitney test) did not show any significant difference between the distributions of *in vitro* sensitivity parameters in males versus females (*p* > 0.05), suggesting that the gender does not influence the cells sensitivity towards PLTX toxicity.

Cell sensitivity data from the 60 healthy volunteers were grouped also on the basis of the blood group, as reported in Figure 3C,D. Similar to the comparison between genders, the non-parametric analysis for unpaired data (Kruskal–Wallis test) followed by the Dunn’s post-test did not show any significant difference between blood groups (*p* > 0.05), considering both EC_50_ and Emax values, suggesting that the blood group does not affect the cells sensitivity towards PLTX toxicity.

Considering the age, a set of correlation analyses between cells sensitivity (EC_50_ and Emax) and the age of each volunteer (*n* = 60) was carried out. However, the non-parametric Spearman’s correlation analysis did not show any significant correlation (*p* > 0.05) between age and EC_50_ or Emax, suggesting that also the age does not influence the cells sensitivity to PLTX.

Overall, these results suggest that demographic variables of the healthy volunteers (gender, age, and blood group) do not affect the cell’s sensitivity to the toxin.

### 2.5. Correlation between In Vitro Cells Sensitivity to PLTX and Gene Expression of Na^+^/K^+^-ATPase Subunits Isoforms

**Figure 4:**
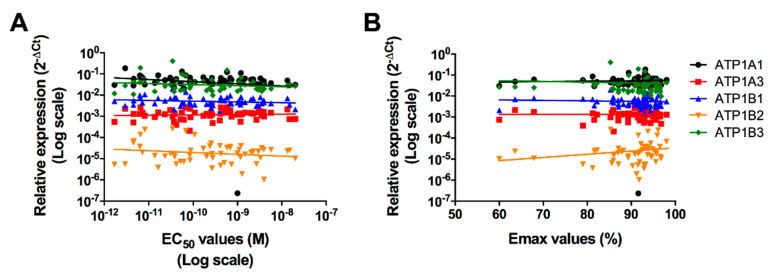
Dispersion graphs of the relative gene expressions of the Na^+^/K^+^-ATPase subunits isoforms recorded in monocytes from the 60 healthy volunteers in function of the EC_50_ (**A**) or the Emax (**B**) values, and the relevant regression lines. Each point represents the mean EC_50_ or Emax values recorded for each volunteer.

To verify whether the expression of each Na^+^/K^+^-ATPase subunit isoform is correlated with cell sensitivity to PLTX, EC_50_, and Emax values from each healthy volunteer were analyzed with respect to each isoform gene expression. The non-parametric Spearman’s correlation analysis did not show any significant correlation between any of the considered isoforms and the EC_50_ values. On the contrary, considering Emax as a parameter of sensitivity to PLTX, a significant positive correlation only between ATP1B2 gene expression and Emax values (rho = 0.30; *p* = 0.025) was recorded, but not for the other isoforms (Figure 4).

**Figure 5:**
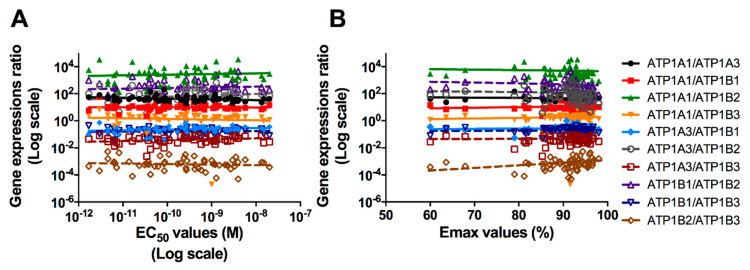
Dispersion graphs of the ratios of relative gene expressions of the different Na^+^/K^+^-ATPase subunits isoforms in function of the EC_50_ (**A**) or the Emax (**B**) values, and relevant regression lines. Each point represents the mean EC_50_ or Emax values recorded for each healthy volunteer.

Finally, the non-parametric Spearman’s correlation analysis was carried out to analyze whether the gene expression ratios of each Na^+^/K^+^-ATPase subunit isoforms may be related to variable cells sensitivity towards the toxin (Figure 5). The analysis showed a significant negative correlation between EC_50_ values and the ATP1A1/ATP1A3 expression ratio (rho = −0.33; *p* = 0.011), a significant positive correlation between EC_50_ values and the ATP1A3/ATP1B3 expression ratio (rho = 0.27; *p* = 0.040) as well as a trend of positive correlation between EC_50_ values and the ATP1A3/ATP1B1 expression ratio (rho = 0.23; *p* = 0.082), even though it is not significant. Considering Emax, the non-parametric Spearman’s correlation analysis determined a significant positive correlation between Emax values and the ATP1B2/ATP1B3 expression ratio (rho = 0.38; *p* = 0.004), a significant negative correlation between Emax values and the ATP1B1/ATP1B2 expression ratio (rho = −0.30; *p* = 0.026) as well as a trend of negative correlation between Emax values and the ATP1A3/ATP1B2 ratio (rho = −0.24; *p* = 0.060), even though it is not significant. These results strengthen the significant role of β2 subunit isoform (ATP1B2) and suggest a secondary role of the α3 subunit isoform (ATP1A3) in modulating the *in vitro* cell sensitivity towards PLTX.

## 3. Discussion

In the omics era, toxicogenetics and toxicogenomics have become a well-established tool to implement conventional toxicological data for the prediction of the actual toxicological impact of a substance on human health [42,43]. However, if toxicogenetics and toxicogenomics are well applied in the risk assessment of pharmaceutical drugs, chemicals and pollutants, they are still underexplored tools in the toxicological evaluation of bioactive marine natural compounds, especially of algal toxins that can accumulate in seafood and induce severe foodborne poisonings in humans. The majority of these toxins displays an extraordinary selectivity towards molecular targets, such as ions channels and ions transporters and receptors, the majority of which are encoded by genes characterized by multiple variants or frequent polymorphisms and mutations. This high variability leads to hypothesize possible genetic-based differences in the individual sensitivity towards these compounds. This aspect should be considered in the risk assessment of these toxins and could help to identify sub-populations at risk of developing severe adverse outcomes. In this view, the present study was carried out as a proof-of-concept applying for the first time an already consolidated experimental design in the toxicogenetics field to PLTX, as one of the most harmful marine compounds known to date.

The documented adverse effects of PLTX in humans are mainly related to oral, inhalation, cutaneous and even ocular exposure. The most severe symptoms, including fatal outcomes, occurred after oral exposure by consumption of contaminated seafood [14,19,20]. Epidemiological data suggest a variable inter-individual sensitivity to the toxic effects of PLTX. Although several factors (i.e., exposure levels, characteristics, and amount of ingested food and/or any concomitant pathology) may influence the toxicity of PLTX, the inter-individual variability in the toxic response could be ascribed also to the involvement of different genetic variants of the Na^+^/K^+^-ATPase, the molecular target of the toxin. These variants lead to the existence of different isoforms of each subunit composing the Na^+^/K^+^-ATPase, which expression is highly variable in a tissue-specific manner, as defined by the Model Organism Protein Expression Database (MOPED) [27]. Moreover, an inter-individual variability in the expression patterns of these isoforms, related also to differences in affinity and sensitivity to cardioactive glycosides, has been demonstrated [31,32,33,34,35]. The variable inter-individual expression pattern of Na^+^/K^+^-ATPase subunits isoforms led us to hypothesize also a variable sensitivity towards PLTX, sharing its molecular target with cardioactive glycosides.

To identify Na^+^/K^+^-ATPase subunits isoforms associated with PLTX sensitivity and identify genetic markers associated with an increased risk of toxic effects in humans, 60 healthy adult volunteers were enrolled in the present study. The peripheral blood monocytes from each volunteer were purified to assess their *in vitro* sensitivity to PLTX by the MTT reduction test, widely used to evaluate the toxin cytotoxicity. Sensitivity parameters were expressed as the toxin concentration inducing 50% of the maximal cytotoxicity (EC_50_) and the maximum cytotoxicity (Emax) induced by the highest concentration of the toxin (10^−7^ M). The recorded EC_50_ and Emax values demonstrate a wide inter-individual variability in the *in vitro* sensitivity towards PLTX. This result corroborates our previous *in vitro* findings using a panel of 9 different cell lines, derived from different tissues and showing a wide range of sensitivity for PLTX binding [44]. However, the present results were obtained using the same cell type (i.e., healthy volunteers’ monocytes), therefore avoiding variable sensitivity due to differences in metabolic pathways and/or signal transduction pathways characterizing different cell types, such as in the previous study.

The genetic analysis of Na^+^/K^+^-ATPase subunits within this study was focused on the α1, α3, β1, β2, and β3 isoforms, excluding the others due to their low or absent expression, in agreement with MOPED data [27] and with a previous study showing these isoforms as the mostly expressed in erythroid precursors isolated from peripheral blood [45]. As expected, relative gene expression data of the subunits isoforms showed a wide inter-individual variability in their gene expression within the 60 enrolled healthy volunteers. To define whether this variability could be dependent on demographic factors, relative gene expression data from each donor were correlated with gender, blood groups, or age. The correlation between gene expression and gender did not highlight any significant result, in contrast to literature data reporting different expression levels of ATP1A1 and ATP1A3 genes in myocardial cells between men and women [30]. However, since gene expression of the Na^+^/K^+^-ATPase isoforms is tissue-specific, it is possible that this discrepancy could be due to the different investigated cell type. Similarly, no significant correlation between gene expression and blood group was recorded, whereas a significant negative correlation was recorded between subjects’ age and ATP1B3 gene expression, suggesting that as the age increases, the expression of the β3 subunit isoform decreases. On the contrary, a previous study carried out on skeletal muscle cells showed an increased β3 subunit isoform expression in humans with average age of 69.4 ± 3.5 years as compared to young people (age mean = 25.5 ± 2.8 years) [46]. Again, such a discrepancy between our findings and literature data could be due to the tissue-specific expression of these genes: Indeed, the level of β3 isoform expression in circulating monocytes is significantly higher than that in muscle cells, such as those of the myocardium (MOPED) [27]. In addition, while our data are based on gene transcription, the conclusion of that study is based on protein expression data.

A similar approach was followed to investigate if the variable monocytes sensitivity to PLTX could be affected by healthy volunteers’ demographic factors. A previous study already demonstrated an increased affinity of the cardioactive glycoside ouabain for lymphocytes Na^+^/K^+^-ATPase in females as compared to males [29]. On the contrary, our results suggest that none of the demographic data (gender, age, and blood group) are related to the variable monocytes sensitivity to PLTX. This discrepancy could be due to the fact that ouabain and PLTX were shown to bind simultaneously to the same Na^+^/K^+^-ATPase, each molecule capable of destabilizing the other, but with different binding sites [26], as supported by a binding study on human keratinocytes [41]. However, the actual binding site of PLTX on Na^+^/K^+^-ATPase has not yet identified, so far, in contrast to that of ouabain, which appears to be located on the α subunits [47]. Hence, a significant role in the cells sensitivity to PLTX could be played by other subunits (i.e., the β ones) or some genetic variants (i.e., different isoforms).

With this in mind, correlations between sensitivity parameters and gene expression data on the Na^+^/K^+^-ATPase isoforms considered in this study were analyzed. In particular, correlation analysis revealed a possible role of the ATP1B2 gene, whose expression appears positively correlated with Emax values. This suggests that a high ATP1B2 gene expression correlates with the increased monocytes sensitivity to PLTX cytotoxicity. Thus, to clarify the possible role of the β2 isoform, we evaluated also the gene expression ratios between the α and β isoforms and their correlation with cell sensitivity to PLTX. Indeed, it is well known that multiple α isoforms can associate with multiple β isoforms in a heterogeneous way to form different α/β subunits combinations in the Na^+^/K^+^-ATPase complex, resulting in different ratios between specific α and β isoforms [48,49]. For instance, different expressions of the α1 isoform, but also of the ratio between α1 isoform and α2 were described in erythrocytes from infants, according to the month of life [50]. The correlation analysis between the isoforms gene expression ratios and sensitivity parameters showed a significant positive correlation between Emax values and the ATP1B2/ATP1B3 gene expression ratio, and a significant negative correlation with the ATP1B1/ATP1B2 gene expression ratio. These results support the hypothesis that the high gene expression of the β2 isoform correlates with increased monocyte sensitivity to the toxin cytotoxicity, in particular when the β2 isoform gene expression is higher than those of the β1 and/or β3 isoforms. This observation highlights once more that the β2 isoform, despite its low relative expression, may represent a genetic marker of monocytes sensitivity to PLTX. In addition, a significant negative correlation between EC_50_ values and ATP1A1/ATP1A3 genes expressions ratio was shown, together with a significant positive correlation between EC_50_ and ATP1A3/ATP1B3 genes expressions ratio. This suggests that a low gene expression of the α3 isoform (as compared to the α1 and/or β3 isoform gene expressions) correlates with an increased cell sensitivity to the toxin. Nonetheless, the possible role of the α3 isoform appears to be minor as compared to that of β2, which is significantly related to the *in vitro* cell sensitivity to PLTX, both when considered alone and in relation to other β isoforms. This observation is of particular interest considering that, as reported above, the α subunit is involved in the binding of cardioactive glycosides to Na^+^/K^+^-ATPase [51,52], with high affinity [31,35]. On the contrary, our results suggest that the gene expression of β subunits (and in particular the isoform β2) is correlated with the high cell sensitivity to PLTX cytotoxic effect. These considerations represent a novel insight on the elucidation of the molecular mechanism of action of the toxin, strengthening the hypothesis that PLTX and ouabain present different binding sites on Na^+^/K^+^-ATPase [26,41], and that the binding site for PLTX might be located on β isoforms. However, further experiments will be necessary to confirm this hypothesis.

In conclusion, the results of this toxicogenetic study showed a direct correlation between gene expression of the β2 subunit isoform of the Na^+^/K^+^-ATPase and the *in vitro* sensitivity of human monocytes to PLTX, shedding light on genetic features underneath the toxin mechanism of action. In particular, individuals with high β2 isoform gene expression levels (both alone and with respect to the β1 and/or β3 isoforms) could be more sensitive to the toxic effects of PLTX. A secondary role could be ascribed to the α3 isoform: subjects with high gene expressions of this isoform compared to the α1 and/or β3 ones could be more sensitive to the toxin effects. To demonstrate that β2 subunit isoform, and to a less extent α3 isoform, are the Na^+^/K^+^-ATPase subunits effectively involved in modulating PLTX cytotoxicity, further studies are needed, including the evaluation of protein expression of Na^+^/K^+^-ATPase subunits and/or PLTX effects toward cells over-expressing the β2 isoform protein. Notwithstanding, this study applied for the first time a toxicogenetic approach to define genetic risk factors influencing the onset of adverse effects in the case of human PLTX poisonings. Given the importance of determining risk factors in case of foodborne poisonings associated with consumption of PLTX contaminated seafood, this study represents a proof-of-concept that can be fruitfully applied to other algal toxins and seafood contaminants, whose molecular targets are already known.

## 4. Materials and Methods

### 4.1. Chemical

PLTX was purchased from Wako Pure Chemicals Industries Ltd. (Osaka, Japan; purity > 90%). All other reagents were of analytical grade and purchased from Sigma-Aldrich (Milan, Italy), if not otherwise specified.

### 4.2. Study Design

Blood samples from 60 healthy adult donors were obtained from the Transfusion Center, Azienda Ospedaliera Universitaria (Trieste, Italy) and immediately processed. From each donor, peripheral blood mononuclear cells (PBMCs) were collected by density gradient centrifugation to characterize their *in vitro* sensitivity to PLTX. In addition, total RNA was extracted to evaluate gene expression for the different isoforms of the Na^+^/K^+^-ATPase α and β subunits, mostly expressed in PBMCs. Gene expressions data were then correlated to those of PBMCs *in vitro* sensitivity to PLTX by means of cytotoxicity.

### 4.3. PBMCs Extraction

Blood samples from healthy adult donors were collected by venepuncture, from April 2017 to March 2018, between 08.00 a.m. and 10.00 a.m. to minimize any response variability due to circadian rhythms. All donors signed an individual review board-approved consent form giving permission for the collection and use of blood for research purposes (WMA Declaration of Helsinki). PBMCs were immediately separated by density gradient centrifugation (600× *g* for 40 min) on Ficoll Paque™ Plus (Healthcare; Milan, Italy), washed twice with phosphate buffer saline (PBS) and re-suspended in RPMI-1640 supplemented with 10% foetal bovine serum (FBS), 1.0 × 10^−2^ M l-Glutamine, 1.0 × 10^−4^ g/mL penicillin, and 1.0 × 10^−4^ g/mL streptomycin. Monocytes were separated from lymphocytes by adhesion: PBMCs were incubated in flat bottom 96-well plates (300.000 PBMCs/well) for 2 h at 37 °C in a humidified 95% air/5% CO_2_ atmosphere, washed with PBS to remove unbound lymphocytes and maintained in culture overnight before treatment with PLTX.

### 4.4. Cytotoxicity Analysis

Healthy donors’ monocytes were exposed to PLTX (10^−13^–10^−7^ M) for 4 h the day after purification from volunteers’ buffy coats. After toxin exposure, cell media were removed and cells were exposed for an additional 4 h to fresh medium containing 0.5 mg/mL 3-(4,5-Dimethyl-2-thiazolyl)-2,5-diphenyl-2H-tetrazolium bromide (MTT). The insoluble crystals were then solubilized by 200 μL/well DMSO and the absorbance was measured by an Automated Microplate Reader EL 311 s (Bio-Tek Instruments, Winooski, VT, USA) at 540/630 nm. The percentage of cell viability was evaluated with respect to negative control (cells not exposed to the toxin) and expressed as mean ± standard error (SE) of 3 independent experiments performed in triplicate for each blood sample of each volunteer. For each healthy donor, cells sensitivity to PLTX was evaluated by means of EC_50_ (toxin concentration giving 50% of the maximal effect, i.e., 50% cell viability reduction) and Emax (percentage of the maximum effect given by the highest toxin concentration; 10^−7^ M) values.

### 4.5. Real Time PCR

Total RNA was extracted from healthy donors’ monocytes using the TRIzol^®^ reagent (Thermo Fisher Scientific; Milan, Italy) according to manufacturer’s instructions. RNA concentration and purity were calculated using a NanoDrop 2000 spectrophotometer (EuroClone; Milan, Italy). The reverse transcription reaction was carried out with the High Capacity RNA-to-cDNA Kit (Thermo Fisher Scientific; Milan, Italy), following the manufacturer’s instructions. Expression levels of ATP1A1, ATP1A3, ATP1B1, ATP1B2 and ATP1B3 genes were evaluated by a SYBR^®^ Green-based (KiCqStart SYBR^®^ Green ReadyMix™; Sigma-Aldrich, Milan, Italy) quantitative real-time PCR (qPCR) following the manufacturer’s instructions and using the CFX96 real-time system-C1000 Thermal Cycler (Bio-Rad Laboratories, Hercules, CA, United States). For ACTB, ATP1A1, ATP1A3, ATP1B1 and ATP1B3 genes, qPCR protocol consisted of an initial denaturation for 3 min at 95 °C, followed by 45 cycles of heating at 95 °C (10 s) and extension at 60 °C (30 s), whereas for ATP1B2, qPCR protocol consisted of an initial denaturation for 3 min at 95 °C, followed by 45 3-step cycles of heating at 95 °C (10 s), 63 °C (15 s) and extension at 72 °C (10 s). Gene expression primers were pre-designed (Table 1) and purchased from Sigma-Aldrich (Milan, Italy). Relative gene expression is quantified by the comparative Ct (cycle threshold) method and represented as 2^−ΔCt^ with respect to the housekeeping beta-actin (ACTB) gene. All the data are the mean ± SE of three experiments performed in duplicate. Amplicon dimension of samples was evaluated using 2% agarose gel electrophoresis to verify the presence of non-specific products that were excluded.

### 4.6. Statistical Analysis

Sensitivity of monocytes from each healthy volunteer to PLTX is expressed as: (i) PLTX concentration inducing 50% of the maximum effect (EC_50_) and (ii) the percentage of the maximum effect (Emax) induced by the highest toxin concentration (10^−7^ M), and are represented as the mean ± standard error (SE) of 3 replicates. Gene expression data for each healthy volunteer are represented as relative expression with respect to the housekeeping ACTB gene, calculated as 2^−ΔCt^ and are the mean ± SE of three independent experiments performed in duplicate. Gene expression values, sensitivity (EC_50_ and Emax), and demographic data of the healthy volunteers (age, blood group) were analysed to determine the median values and the relative percentile range (GraphPad software, version 6.0). Data were analysed by Spearman’s non-parametric correlation analysis (GraphPad software, version 6.0) since they did not result in a Gaussian distribution (D’Agostino and Pearson omnibus normality test; GraphPad software, version 6.0) even after logarithmic normalization. Significant correlations were defined for *p* values < 0.05. Gene expression and sensitivity values were also grouped on the basis of the demographic data of healthy volunteers (gender and blood group) and analyzed by non-parametric analysis for unpaired data (Mann–Whitney test and Kruskal–Wallis test followed by Dunn’s post-test, respectively) and defined significant for *p* values < 0.05 (GraphPad software, version 6.0; GraphPad Software; San Diego, CA, USA).

## Figures and Tables

**Table 1:** Pre-designed primers (Sigma-Aldrich; Milan, Italy) for qPCR analysis of genes encoding for the different isoforms of α (1 and 3) and β (1, 2 and 3) subunits of the Na^+^/K^+^ ATPase.

Gene	Forward Primer (5′→3′)	Reverse Primer (5′→3′)
ACTB	5′-GACGACATGGAGAAAATCTG	5′-ATGATCTGGGTCATCTTCTC
ATP1A1	5′-GTGTCTTTCTTCATCCTTTCTC	5′-CCACAGCTTCTAAGTTCTTC
ATP1A3	5′-ATCTGCTCAGATAAGACAGG	5′-AACTCTTGTCAAATGAGGTC
ATP1B1	5′-AAAGTACAAAGATTCAGCCC	5′-CATGATTAAAGTCTCCTCGTTC
ATP1B2	5′-CTCATGTACTTCCCCTACTATG	5′-ATGCGACATTCTACATTCAC
ATP1B3	5′-GCCAAGGATAGATTGTGTTTC	5′-CTGTTACTTCTTTCCCAGTG

## Supplementary Materials

Supplementary materials can be found at https://www.mdpi.com/1422-0067/21/16/5833/s1.

## References

[1] Moore R.E., Scheuer P.J. (1971). Palytoxin: A new marine toxin from a coelenterate. Science.

[2] Ukena T., Satake M., Usami M., Oshima Y., Naoki H., Fujita T., Kan Y., Yasumoto T. (2001). Structure elucidation of ostreocin D, a palytoxin analog isolated from the dinoflagellate ostreopsis siamensis. Biosci. Biotechnol. Biochem..

[3] Lenoir S., Ten-Hage L., Turquet J., Quod J.P., Bernard C., Hennion M.C. (2004). First evidence of palytoxin analogues from an *Ostreopsis mascarenensis* (Dinophyceae) bentic bloom in Southwestern Indian Ocean. J. Phycol..

[4] Ciminiello P., Dell’Aversano C., Fattorusso E., Forino M., Tartaglione L., Grillo C., Melchiorre N., Aversano C.D. (2008). Putative palytoxin and its new analogue, ovatoxin-a, in *Ostreopsis ovata* collected along the ligurian coasts during the 2006 toxic outbreak. J. Am. Soc. Mass Spectrom..

[5] Rossi R., Castellano V., Scalco E., Serpe L., Zingone A., Soprano V. (2010). New palytoxin-like molecules in Mediterranean *Ostreopsis* cf. ovata (dinoflagellates) and in Palythoa tuberculosa detected by liquid chromatography-electrospray ionization time-of-flight mass spectrometry. Toxicon.

[6] Amzil Z., Sibat M., Chomérat N., Grossel H., Marco-Miralles F., Lemée R., Nézan E., Sechet V. (2012). Ovatoxin-a and palytoxin accumulation in seafood in relation to *Ostreopsis cf. ovata* blooms on the French Mediterranean coast. Mar. Drugs.

[7] Brissard C., Herrenknecht C., Sechet V., Hervé F., Pisapia F., Harcouet J., Lemée R., Chomérat N., Hess P., Amzil Z. (2014). Complex toxin profile of French Mediterranean *Ostreopsis cf. ovata* strains, seafood accumulation and ovatoxins prepurification. Mar. Drugs.

[8] García-Altares M., Tartaglione L., Carnicer O., De La Iglesia P., Forino M., Diogène J., Ciminiello P., Dell’Aversano C. (2014). The novel ovatoxin-g and isobaric palytoxin (so far referred to as putative palytoxin) from *Ostreopsis* cf. ovata (NW Mediterranean Sea): Structural insights by LC-high resolution MSn. Anal. Bioanal. Chem..

[9] Gladan Z.N., Arapov J., Casabianca S., Penna A., Honsell G., Brovedani V., Pelin M., Tartaglione L., Sosa S., Dell’Aversano C. (2019). Massive occurrence of the harmful benthic dinoflagellate *Ostreopsis* cf. ovata in the Eastern Adriatic Sea. Toxins.

[10] Soliño L., García-Altares M., Godinho L., Costa P.R. (2020). Toxin profile of *Ostreopsis* cf. ovata from Portuguese continental coast and Selvagens Islands (Madeira, Portugal). Toxicon.

[11] Kerbrat A.S., Amzil Z., Pawlowiez R., Golubic S., Sibat M., Darius H.T., Chinain M., Laurent D. (2011). First evidence of palytoxin and 42-hydroxy-palytoxin in the marine cyanobacterium trichodesmium. Mar. Drugs.

[12] Durando P., Ansaldi F., Oreste P., Moscatelli P., Marensi L., Grillo C., Gasparini R., Icardi G., Collaborative Group for the Ligurian Syndromic Algal Surveillance (2007). Ostreopsis ovate and human health: Epidemiological and clinical features of respiratory syndrome outbreaks from a two-year syndromic surveillance, 2005-06, in north-west Italy. Euro Surveill..

[13] Tichadou L., Glaizal M., Armengaud A., Grossel H., Lemée R., Kantin R., Lasalle J.L., Drouet G., Rambaud L., Malfait P. (2010). Health impact of unicellular algae of the *Ostreopsis* genus blooms in the Mediterranean Sea: Experience of the French Mediterranean coast surveillance network from 2006 to 2009. Clin. Toxicol..

[14] Tubaro A., Durando P., Del Favero G., Ansaldi F., Icardi G., Deeds J., Sosa S. (2011). Case definitions for human poisonings postulated to palytoxins exposure. Toxicon.

[15] Rhodes L.L. (2011). World-wide occurrence of the toxic dinoflagellate genus *Ostreopsis* Schmidt. Toxicon.

[16] Del Favero G., Sosa S., Pelin M., D’Orlando E., Florio C., Lorenzon P., Poli M., Tubaro A. (2012). Sanitary problems related to the presence of Ostreopsis spp. in the Mediterranean Sea: A multidisciplinary scientific approach. Annali dell’Istituto Superiore di Sanità.

[17] Deeds J.R., Handy S.M., White K.D., Reimer J.D. (2011). Palytoxin found in *Palythoa* sp. zoanthids (Anthozoa, Hexacorallia) sold in the home aquarium trade. PLoS ONE.

[18] Pelin M., Brovedani V., Sosa S., Tubaro A. (2016). Palytoxin-containing aquarium soft corals as an emerging sanitary problem. Mar. Drugs.

[19] Deeds J.R., Schwartz M.D. (2010). Human risk associated with palytoxin exposure. Toxicon.

[20] Patocka J., Nepovimova E., Wu Q., Kuca K. (2017). Palytoxin congeners. Arch. Toxicol..

[21] Noguchi T., Hwang D.F., Arakawa O., Daigo K., Sato S., Ozaki H., Kawai N., Ito M., Hashimoto K., Gopalakrishnakone P., Tam C.K. (1987). Palytoxin as a causative agent in the parrotfish poisoning. Progress in Venom and Toxin Research: Proceedings of the First Asia-Pacific Congress on Animal, Plant and Microbial Toxins.

[22] Onuma Y., Satake M., Ukena T., Roux J., Chanteau S., Rasolofonirina N., Ratsimaloto M., Naoki H., Yasumoto T. (1999). Identification of putative palytoxin as the cause of clupeotoxism. Toxicon.

[23] Taniyama S., Mahmud Y., Terada M., Takatani T., Arakawa O., Noguchi T. (2002). Occurrence of a food poisoning incident by palytoxin from a serranid *Epinephelus* sp. in Japan. J. Nat. Toxins.

[24] Wieringa A., Bertholee D., Ter Horst P., Brand I.V.D., Haringman J., Ciminiello P. (2014). Respiratory impairment in four patients associated with exposure to palytoxin containing coral. Clin. Toxicol..

[25] Hilgemann D.W. (2003). From a pump to a pore: How palytoxin opens the gates. Proc. Natl. Acad. Sci. USA.

[26] Artigas P., Gadsby D.C. (2004). Large diameter of palytoxin-induced Na/K pump channels and modulation of palytoxin interaction by Na/K pump ligands. J. Gen. Physiol..

[27] Higdon R., Stewart E., Stanberry L., Haynes W., Choiniere J., Montague E., Anderson N., Yandl G., Janko I., Broomall W. (2013). MOPED enables discoveries through consistently processed proteomics data. J. Proteome Res..

[28] Glorioso N., Herrera V.L., Bagamasbad P., Filigheddu F., Troffa C., Argiolas G., Bulla E., Decano J.L., Ruiz-Opazo N. (2007). Association of ATP1A1 and dear single-nucleotide polymorphism haplotypes with essential hypertension: Sex-specific and haplotype-specific effects. Circ. Res..

[29] Scarrone S., Balestrino M., Frassoni F., Pozzi S., Gandolfo C., Podestà M., Cupello A. (2007). Sex differences in human lymphocyte Na,K-ATPase as studied by labeled ouabain binding. Int. J. Neurosci..

[30] Gaborit N., Varró A., Le Bouter S., Szüts V., Escande D., Nattel S., Demolombe S. (2010). Gender-related differences in ion-channel and transporter subunit expression in non-diseased human hearts. J. Mol. Cell. Cardiol..

[31] Hauck C., Potter T., Bartz M., Wittwer T., Wahlers T., Mehlhorn U., Scheiner-Bobis G., McDonough A.A., Bloch W., Schwinger R.H. (2009). Isoform specificity of cardiac glycosides binding to human Na+,K+-ATPase alpha1beta1, alpha2beta1 and alpha3beta1. Eur. J. Pharmacol..

[32] Katz A., Lifshitz Y., Bab-Dinitz E., Kapri-Pardes E., Goldshleger R., Tal D.M., Karlish S.J.D. (2010). Selectivity of digitalis glycosides for isoforms of human Na,K-ATPase. J. Boil. Chem..

[33] Shibuya K., Fukuoka J., Fujii T., Shimoda E., Shimizu T., Sakai H., Tsukada K. (2010). Increase in ouabain-sensitive K+-ATPase activity in hepatocellular carcinoma by overexpression of Na+,K+-ATPase α3-isoform. Eur. J. Pharmacol..

[34] Weigand K.M., Laursen M., Swarts H.G.P., Engwerda A.H.J., Prüfert C., Sandrock J., Nissen P., Fedosova N.U., Russel F.G.M., Koenderink J.B. (2014). Na+,K+-ATPase isoform selectivity for digitalis-like compounds is determined by two amino acids in the first extracellular loop. Chem. Res. Toxicol..

[35] Lev M.C., Karlish S.J.D., Garty H. (2015). Cardiac glycosides induced toxicity in human cells expressing α1, α2, or α3-isoforms of Na-K-ATPase. Am. J. Physiol. Physiol..

[36] Habermann E., Chhatwal G.S. (1982). Ouabain inhibits the increase due to palytoxin of cation permeability of erythrocytes. Naunyn-Schmiedeberg’s Arch. Pharmacol..

[37] Schilling W.P., Snyder D., Sinkins W.G., Estacion M. (2006). Palytoxin-induced cell death cascade in bovine aortic endothelial cells. Am. J. Physiol. Physiol..

[38] Vale-Gonzalez C., Pazos M., Alfonso A., Vieytes M., Botana L. (2007). Study of the neuronal effects of ouabain and palytoxin and their binding to Na,K-ATPases using an optical biosensor. Toxicon.

[39] Pelin M., Zanette C., De Bortoli M., Sosa S., Della Loggia R., Tubaro A., Florio C. (2011). Effects of the marine toxin palytoxin on human skin keratinocytes: Role of ionic imbalance. Toxicology.

[40] Pelin M., Sosa S., Della Loggia R., Poli M., Tubaro A., Decorti G., Florio C. (2012). The cytotoxic effect of palytoxin on Caco-2 cells hinders their use for in vitro absorption studies. Food Chem. Toxicol..

[41] Pelin M., Boscolo S., Poli M., Sosa S., Tubaro A., Florio C. (2013). Characterization of palytoxin binding to HaCaT cells using a monoclonal anti-palytoxin antibody. Mar. Drugs.

[42] Gomase V.S., Tagore S. (2008). Toxicogenomics. Curr. Drug. Metab..

[43] Liu Z., Huang R., Roberts R., Wu L. (2019). Toxicogenomics: A 2020 Vision. Trends Pharmacol. Sci..

[44] Pelin M., Sosa S., Brovedani V., Fusco L., Poli M., Tubaro A. (2018). A novel sensitive cell-based immunoenzymatic assay for palytoxin quantitation in mussels. Toxins.

[45] Hoffman J.F., Wickrema A., Potapova O., Milanick M., Yingst D.R. (2002). Na pump isoforms in human erythroid progenitor cells and mature erythrocytes. Proc. Natl. Acad. Sci. USA.

[46] Wyckelsma V.L., McKenna M.J. (2016). Effects of age on Na+,K+-ATPase expression in human and rodent skeletal muscle. Front. Physiol..

[47] Keenan S.M., Delisle R.K., Welsh W.J., Paula S., Ball W.J. (2005). Elucidation of the Na+, K+-ATPase digitalis binding site. J. Mol. Graph. Model..

[48] Wang J., Schwinger R.H., Frank K., Muller-Ehmsen J., Martín-Vasallo P., Pressley T.A., Xiang A., Erdmann E., McDonough A.A. (1996). Regional expression of sodium pump subunits isoforms and Na+-Ca+ exchanger in the human heart. J. Clin. Investig..

[49] Clausen M.V., Hilbers F., Poulsen H. (2017). The structure and function of the Na,K-ATPase isoforms in Health and disease. Front. Physiol..

[50] Vasarhelyi B., Vér A., Nobilis A., Szabo T., Tulassay T. (1998). Functional and structural properties of Na+/K+-ATPase enzyme in neonatal erythocytes. Eur. J. Clin. Investig..

[51] Scheiner-Bobis G., Schneider H. (1997). Palytoxin-induced channel formation within the Na+/K+-ATPase does not require a catalytically active enzyme. Eur. J. Biochem..

[52] Diederich M., Muller F., Cerella C. (2017). Cardiac glycosides: From molecular targets to immunogenic cell death. Biochem. Pharmacol..

