# Supplementary material for: In Vitro Cell Sensitivity to Palytoxin Correlates with High Gene Expression of the Na+/K+-ATPase β2 Subunit Isoform
Source: Int J Mol Sci. 2020 Aug 14;21(16):5833. doi: 10.3390/ijms21165833 (PMC7461505; doi:10.3390/ijms21165833)
Supplement: Supplementary file 1 [file ijms-21-05833-s001.pdf]

Supplementary Materials

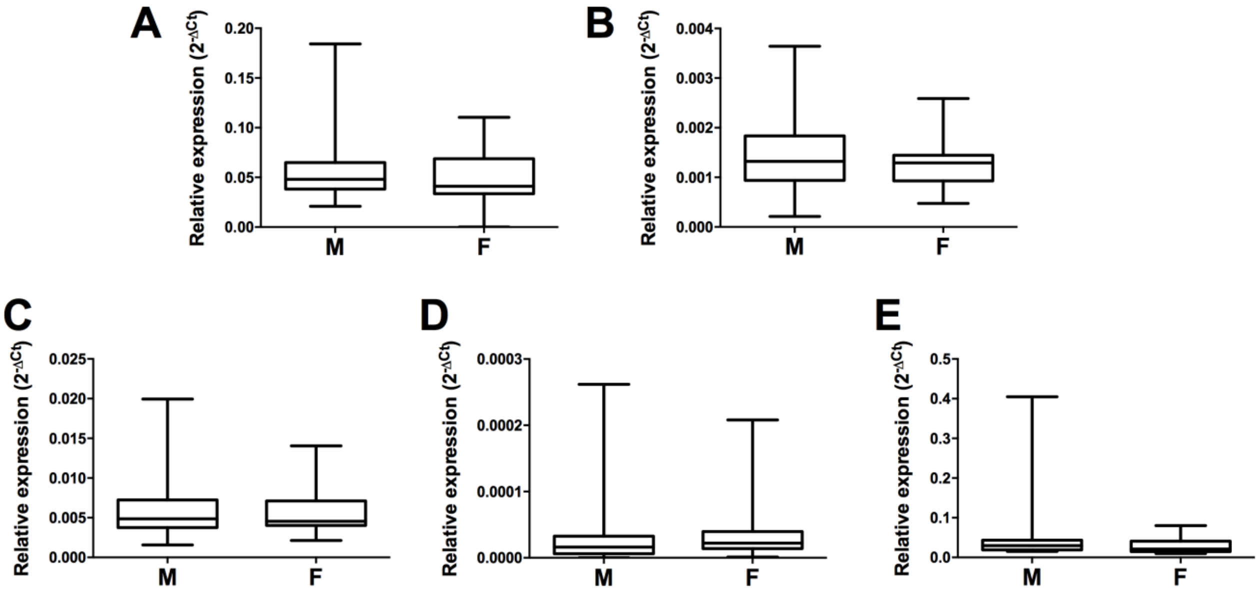

Figure S1. Distribution of relative gene expressions of the single Na^+/K^+-ATPase isoforms grouped by the gender (male = 41; female = 19), calculated as $2^{-\Delta Ct}$ with respect to the housekeeping ACTB gene: (**A**) α1 isoform (ATP1A1); (**B**) α3 isoform (ATP1A3); (**C**) β1 isoform (ATP1B1); (**D**) β2 isoform (ATP1B2); (**E**) β3 isoform (ATP1B3). For each volunteer, relative expression data were the means of three experiments performed in duplicate.

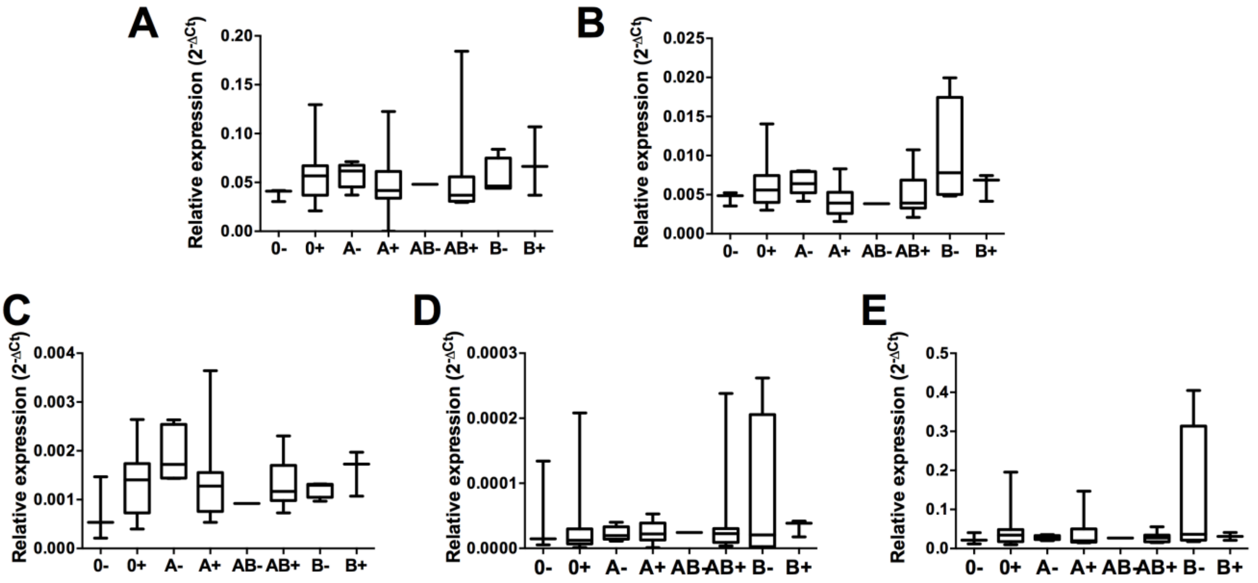

Figure S2. Distribution of relative gene expressions of the single Na^+/K^+-ATPase isoforms grouped by the blood group, calculated as $2^{-\Delta Ct}$ with respect to the housekeeping ACTB gene (0^- = 3; 0^+ = 22; A^- = 5; A^+ = 12; AB^- = 1; AB^+ = 10; B^- = 4; B^+ = 3): (**A**) α1 isoform (ATP1A1); (**B**) α3 isoform (ATP1A3); (**C**) β1 isoform (ATP1B1); (**D**) β2 isoform (ATP1B2); (**E**) β3 isoform (ATP1B3). For each volunteer, relative expression data were the means of three experiments performed in duplicate.
